# The clustering of multiple health and lifestyle behaviors among Swedish adolescents: a person-oriented analysis

**DOI:** 10.3389/fpubh.2023.1178353

**Published:** 2023-07-19

**Authors:** Kenisha Russell Jonsson, Maria Corell, Petra Löfstedt, Nicholas Kofi Adjei

**Affiliations:** ^1^School of Public Health and Community Medicine, Institute of Medicine, Gothenburg University, Göteborg, Sweden; ^2^Department of Public Health, Policy and Systems, University of Liverpool, Liverpool, United Kingdom; ^3^Leibniz Institute for Prevention Research and Epidemiology–BIPS, Bremen, Germany; ^4^Health Sciences Bremen, University of Bremen, Bremen, Germany

**Keywords:** health behaviors, lifestyle, alcohol, diet, physical activity, smoking, sleep, children and adolescents

## Abstract

**Background:**

Knowledge of the distribution, prevalence, and clustering of multiple health and lifestyle related behaviors (HLBs) among adolescents can inform the development of effective health-promoting policies and interventions. We assessed the clustering of multiple HLBs among 11, 13 and 15-year-old Swedish adolescents and examined the socioeconomic and demographic correlates for the identified clusters.

**Methods:**

We used data from the 2017/2018 Swedish Health Behaviour in School-aged children (HBSC) study to conduct sex and age-stratified latent class analysis (LCA). The LCA was based on five HLBs: eating behavior and habits (EBH), physical activity (PA), tobacco usage (TU), alcohol consumption (AC) and sleeping habits and patterns (SHPs). Multinomial logistic regression models were used to assess the associations between the identified clusters and the socioeconomic and demographic characteristics of adolescents and their parents.

**Results:**

Health behaviors varied by sex and age. Four distinct clusters were identified based on sex: cluster 1 (Mixed eating behaviors and habits, physical activity and low alcohol consumption), cluster 2 (Healthy lifestyle behaviors), cluster 3 (Unhealthy lifestyle behaviors), and cluster 4 (Breakfast, low alcohol consumption and tobacco usage). In the age-stratified analyzes, three clusters were identified: cluster 1 (Unhealthy lifestyle behaviors), cluster 2 (Moderately healthy lifestyle behaviors) and cluster 3 (Healthy lifestyle behaviors). The multinomial analysis showed that sex, age, family situation and perceived family wealth were strong predictors of health behaviors. Unhealthy behaviors were most commonly associated with socioeconomic disadvantage, having a migrant background, and living in reconstructed families or single-parent households.

**Conclusion:**

Health behaviors vary significantly based on socioeconomic and demographic circumstances. Targeted policies and intervention programs are necessary to improve HLBs among vulnerable and at-risk adolescents.

## Introduction

Health and lifestyle behaviors (HLBs) in adolescence, such as eating behavior and habits (EBH), physical activity (PA), tobacco usage (TU), alcohol consumption (AC) and sleeping habits and patterns (SHP) may have both short and long-term health consequences (1–5). In Sweden, there are public health concerns about the impact of HLBs on adolescents physical and mental health (6, 7). Adolescence is a critical developmental stage and an important life stage where HLBs and inequalities are shaped (8–12). Prior evidence suggests that HLBs formed during adolescence persist into adulthood (8–12). Engaging in unhealthy lifestyle behaviors increases the immediate risks of poor physical and mental health problems (1, 4, 6, 13–15), but also elevates the risk of morbidity and mortality in adulthood (2, 3, 16).

Poor HLBs have been linked to significant increases in non-communicable diseases (NCDs) such as obesity, diabetes, cancer and heart diseases (17). NCDs are known contributors to the burden of disease and premature death among adults in Sweden [13]. They impose significant costs on both individuals and society, particularly on the health and care systems [18]. Moreover, research has shown that EBH, PA, TU, AC and SHP tend to co-occur or cluster in complex ways (19, 20), and that the combination of these behaviors may have more conducive or detrimental effects on an individual’s health, than the effects of single HLBs (21). The literature further suggests that the effects of HLBs may be multiplicative and cumulative rather than additive (21). Previous studies from high income countries have shown that HLBs tend to cluster among people with similar socioeconomic and demographic characteristics (20–24). Notably, individuals from lower socioeconomic backgrounds have been associated with engaging in less healthy behaviors (20, 22–25).

Socioeconomic inequalities in health have been identified in Sweden (26, 27), suggesting that patterns of health behaviors may cluster differently based on socioeconomic and demographic factors. Therefore, understanding how multiple HLBs cluster is crucial in order to develop appropriate public health strategies and interventions (19, 28). However, the clustering of health behaviors among adolescents, and the socioeconomic and demographic determinants of multiple HLBs among adolescents is understudied (24, 29), leading to a knowledge gap. This study will therefore contribute to the literature by assessing the patterning of multiple HLBs in the Swedish context.

### Research aims

The aim of the study was twofold. Firstly, the study aimed to assess the clustering of five health and lifestyle behaviors [eating behavior and habits (EBH), physical activity (PA), tobacco usage (TU), alcohol consumption (AC) and sleeping habits and patterns (SHP)] among Swedish adolescents aged 11, 13 and 15 stratified by sex and age. Secondly, we aimed to investigate the associations between the identified clusters and the socioeconomic and demographic characteristics of adolescents and their parents (i.e., sex, age, migrant background, family situation, perceived family wealth and family affluence).

## Methods

### Data and sample

The analyzes were based on a nationally representative, cross-sectional sample of adolescents from the 2017/2018 Swedish component of the Health Behavior in School-Aged Children (HBSC) survey. It is a World Health Organization (WHO) collaborative study (30), conducted by the Public Health Agency of Sweden and Statistics Sweden. It is a school based study, conducted during school hours. Participation is voluntary and anonymous. The Swedish HBSC participants were selected based on a two-stage cluster design. First, a nationally representative sample of 450 schools were selected, and in the second stage, a single class from each school (5th graders ≈ 11 year olds, 7th graders ≈13 year olds, 9 graders ≈ 15 year olds) was randomly selected to participate. All students in the selected classes were then invited to participate in the survey. The final sample included 4,215 students from 213 schools, with a response rate of 89% in the participating schools, consisting of 2,114 girls (50.2%) and 2,101 boys (49.8%).

### Health and lifestyle behavior measures

#### Eating behaviors and habits

We assessed EBH using seven individual items that captured the regularity of breakfast and the frequency of intake of four food items among adolescents. The first two items assessed the regularity of breakfast consumption on school days and weekends/non-school days. Respondents were asked to estimate the number of weekdays/weekends they had breakfast (more than having than a glass of milk or fruit juice). The responses provided were dichotomized as every day (5 days a week) vs. less than every day (do not consume breakfast on a daily basis). Similarly, breakfast on weekends/non-school days were coded as every day (both Saturday and Sunday) vs. less than every day (never or one of the two days). This coding is in line with how these variables have been treated in other studies using HBSC data (31, 32). Additionally, four items were used to assess the frequency of intake per week, for: (1) fruits, (2) vegetables, (3) sugary soft drinks, and (4) sweets (including chocolate). Participants could choose from the following response options: “never,” “less than once a week,” “once a week,” “two to four times a week,” “five to six times a week,” “once a day,” or “more than once a day.” The consumption of fruits and vegetables was then categorized as “at least once a day” or “less than once a day.” The consumption of sugary soft drinks and sugary foods was categorized as “once a week or less” or “more than once a week.”

EBH was operationalized to ensure comparability with international HBSC reporting standards (31, 32). The exception is sugary soft drinks and sweets, which were coded to reflect Swedish traditions of Saturday candy ‘lördagsgodis’ and cozy Fridays ‘fredagsmys’, where families indulge in these dietary behaviors (33).

#### Physical activity

PA was captured with responses to the question, “how many days in the past week were you physically active for 60 minutes or more?,” and this was exemplified as any activity that increased the heart rate and breathlessness. The responses provided ranged from 0–7 days. From this, two separate measures were coded. First, *Low PA* was operationalized as respondents who reported engaging in at least 1 hour of physical activity but on fewer than 3 days during the week. The second measure categorized respondents as having high PA if they engaged in physical activity for at least 1 hour per day, 7 days per week were categorized as having high PA. In accordance with the WHO physical activity recommendations (34), these items reflect low respective sufficient physical activity levels for an adolescent. In addition to information on General PA, the HBSC study also collects data on adolescents’ participation in any sports-related activities outside of school hours, i.e., during leisure time. This was assessed with the question, “Outside of school hours, how many days per week do you usually exercise in your free time so much that you get out of breath or sweat?” The possible responses ranged from 1 (every day) to 7 (never). This was dichotomized to create a measure of *Vigorous Physical Activity (VPA)* indicating adolescents engaged sports-related PA at least 4 times per week vs. those engaging in sports-related PA less than 4 times per week. This was coded in line with international standards and previous usage (34, 35).

#### Tobacco use

Tobacco usage (TU) was assessed with two questions asking “how many days (if any) have you smoked cigarettes in your life?” and “how many days (if any) have you used snus/snuff in your life?’. Possible responses ranged from 1 (never) to 7 (30 days or more) (32). The responses from both were re-categorized into a single dichotomous measure with responses coded 1 to indicate respondents who had never engaged in TU compared to those who had previously used at least one of these tobacco products (no tobacco usage vs. tobacco usage).

#### Alcohol consumption

Three questions were used to measure the prevalence of AC including drunkenness and binge drinking among adolescents (36). First, respondents were asked “how many days (if any) have you drunk alcohol in your life?,” and provided with responses ranging from 1 (never) to 7 (30 days or more). This was recoded as a dichotomous indicator never drank alcohol vs. drank previously (≥1). Two additional questions were created from responses to the question “have you ever drunk so much alcohol that you got really drunk… (i) in your life (lifetime prevalence) or (ii) during the last 30 days (current prevalence)?” Possible responses ranged from 1 (never) to 7 (more than 10 times). Two dichotomous variables were then created, the first indicating no binge drinking vs. binge drinking (≥1), which is a high frequency of AC during the last 30 days, and the second indicating whether the respondent had never been drunk vs. drunkenness (≥1) at any time previously.

#### Sleeping habits and patterns

SHPs on school days and weekends/holidays (non-school days) were based on four items asking respondents: “When do you usually go to bed?” and “When do you usually wake up?” Bedtimes ranged in half-hour intervals from “No later than 21:00” to “2:00 or later” for school days, and to “4:00 or later” on non-school days. Waketimes ranged in half-hour intervals from “No later than 5:00” to “8:00 or later” for school days and from “No later than 7:00” to “14:00 or later” on non-school days. Sleep duration for school and non-school days was calculated as the difference between bedtimes and waketimes. For responses at the ends of the scale, we used the minimum/maximum stated time (e.g., 14:00 if the waketime response was “14:00 or later” and 5:00 if the waketime response was “No later than 5:00”). We assessed whether adolescents in the sample slept 9 h or more based on current international guidelines recommending that school-aged children sleep between 9–11 h and adolescents 8–10 h (37, 38).

### Covariates

Socioeconomic circumstances was assessed using (a) *Perceived family wealth,* which is a relative measure of wealth, based on the adolescent’s perception of their family’s economic situation. This was categorized as: (1) quite or very well off, (2) average, (3) not so well off/not at all well off; (b) The *Family Affluence Scale* (FAS) is a widely used and validated absolute measure of wealth (39). It is scored using six items (range: 0–13), based on a family’s material possessions and lifestyle including number of: cars (0, 1, 2 or more); bathrooms (0, 1, 2, 3 or more), computers (0, 1, 2, > 2); and holidays abroad during the last 12 months (0, 1, 2, 3 or more); and whether they shared a bedroom (no/yes); or had a dishwasher (no/yes). The sum is then converted into a so-called “ridit-based score” and is used to divide the study participants into quintiles. Adolescents in the lowest quintile were categorized low FAS (equivalent to a score of 0–7), the middle three quintiles were categorized as medium FAS(equivalent to a score of 8–11) and the highest quintile were categorized as having high FAS (equivalent to a score of 12–13) (39). *Family situation* described the composition of each household and was categorized to include adolescents residing (a) with both parents; (b) in reconstructed families (including step-parents); (c) single parent households and (d) in foster/children’s home or with other caregivers. *Migrant background* was categorized to indicate respondents who were foreign born, those born in Sweden and the migrant status of their parents, that is, whether one or both parents were born abroad. Finally, sex and the three sampled age groups (32) were used to stratify the analyzes.

### Analytical strategy

First, multiple clusters of HLBs were identified by estimating a series of Latent class analysis (LCA) using PROC LCA command (SAS version 1.3.2) (40). Five successive sex and age-stratified models of 2 to 6 clusters were estimated separately. One thousand iterations of each model using random starting values were fitted to ensure model identification, missing data was assumed to be at random and handled within the EM algorithm. G^2^ frequencies were compared and the solution with the most common and lowest G^2^ value was identified as reaching a maximum likelihood solution (40). Statistical fit indices including the Akaike Information Criterion (AIC) (41) and Bayesian Information Criterion (BIC) (42) were examined to determine the number of clusters that best fit the data. Low AIC and BIC values are generally deemed to be good fitting models (40). In addition, the appropriate number of clusters were chosen based on conceptual considerations, theory, cluster distinctiveness and interpretability (40).

The second step of the analysis consisted of conducting multinomial logistic regression to assess the socioeconomic and demographic correlates of each identified cluster while taking into account classification uncertainty in each latent cluster. The likelihood of cluster membership were described as risk ratios (RR) with 95% confidence interval (CI). Analyzes were conducted in Stata version 16.1.

## Results

### Descriptive characteristics of the sample

Table 1 shows the descriptive characteristics of the final sample (*N* = 3,937). The overall distribution was fairly equal by sex (Girls: 51% vs. Boys: 49%). The proportion of respondents increased by age: Approximately 28.2% of the sample were 11-year-olds, 33.8% were 13-year-olds and 38.0% were 15-year-olds. Overall, the mean age was 13.2 years (SD = 1.6). There were notable differences in the proportion of adolescents living with both parents (64.8%), compared to those living in reconstructed families (12.9%), single parent households (7.1%) or foster/children’s home or other caregivers (4.3%). Regarding migrant background, 10.2% of the sample were foreign born while the majority were born in Sweden 64.6% and of those born in Sweden 12.7% had one parent with a migrant background and 12.5% had both parents with a migrant background. The distribution of FAS indicated that approximately 17.5% of respondents were classified as having high affluence, the majority were classified as having medium affluence (59.4%) and 20.0% as having low affluence. Regarding perceived family wealth, the majority perceived their family as being quite or very well off (80.7%). Approximately 15% perceived their family as having average wealth and 2.5% as not at all/not so well off.

**Table 1:** Socioeconomic and demographic characteristics of the sample.

	*N*	Percent
Sex		
Boys	1939	49.25
Girls	1998	50.75
Age		
11	1,110	28.19
13	1,330	33.78
15	1,497	38.02
Family situation		
Both parents	2,551	64.80
Reconstructed Family	507	12.88
Single parent	280	7.11
Foster/children’s home or other	169	4.29
Missing	430	10.92
Migrant background		
Swedish born	2,542	64.57
Foreign born	402	10.21
One Parent	501	12.73
Both Parents	492	12.50
Family Affluence Scale (FAS)		
High affluence	689	17.50
Middle affluence	2,338	59.39
Low affluence	789	20.04
Missing	121	3.07
Perceived family wealth		
Fairly or very well off financially	3,177	80.70
Average financially	579	14.71
Not at all/not so well off	97	2.46
Missing	84	2.13

2017/18 Swedish HBSC study, authors analysis.

### Model testing and selection

Analyzes for the total dataset indicated that there were better fitting models for identifying distinct clusters by sex and age separately (Full results not shown). Hence, model testing was conducted separately by sex and age, with sequential analysis of nested (constrained and unconstrained) models for measurement invariance (MI). Model fit indices are presented for sex and age separately (Supplementary Table S1). All participants were assigned to the cluster in which they had the highest probability of membership, which were well-identified, interpretable, and conceptually meaningful (29).

#### Comparison of the clusters by sex

We identified four distinct clusters based on prominent health-related behaviors (HLBs): Cluster 1 - “Mixed EBH, PA, and low AC,” Cluster 2 - “Healthy lifestyle behaviors,” Cluster 3 - “Unhealthy lifestyle behaviors,” and Cluster 4 - “Breakfast, low AC, and TU.” When considering cluster membership probabilities by sex, girls had a higher proportion (27.9%) in the healthy lifestyle behaviors cluster compared to boys (21.5%). Conversely, boys were more represented in the other three clusters: mixed EBH, PA, and low AC (31% boys vs. 28.4% girls), unhealthy lifestyle behaviors (12.9% boys vs. 9.9% girls), and breakfast, low AC, and TU (34.7% boys vs. 33.8% girls).

**Figure 1:**
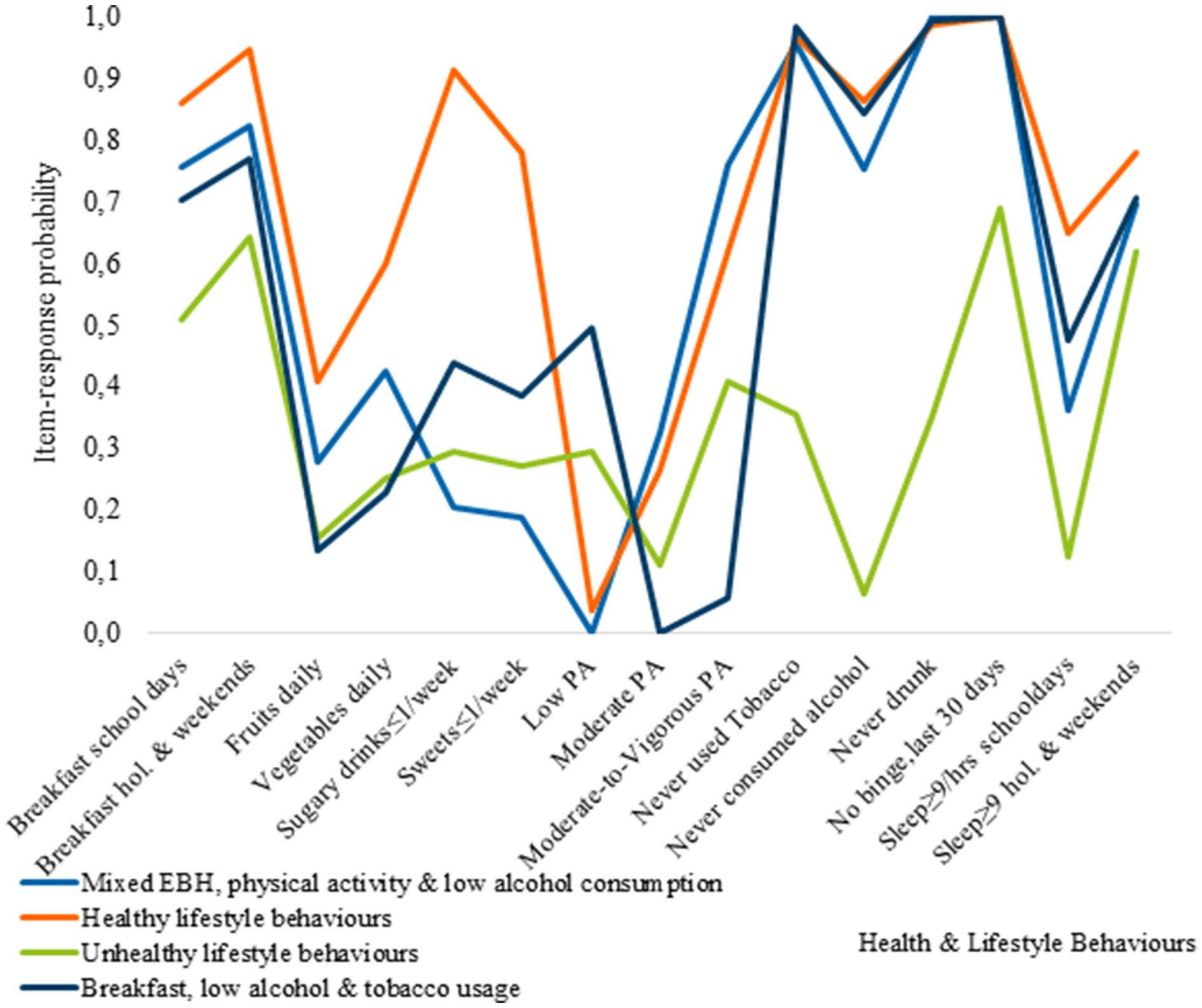
Item-response probabilities across each of the clusters for boys.

Figures 1 2 presents the item-response probabilities, indicating the likelihood for boys’ respective girls to engage in fifteen HLBs. These probabilities are determined by the underlying patterns of responses, which in turn define each cluster.

**Figure 2:**
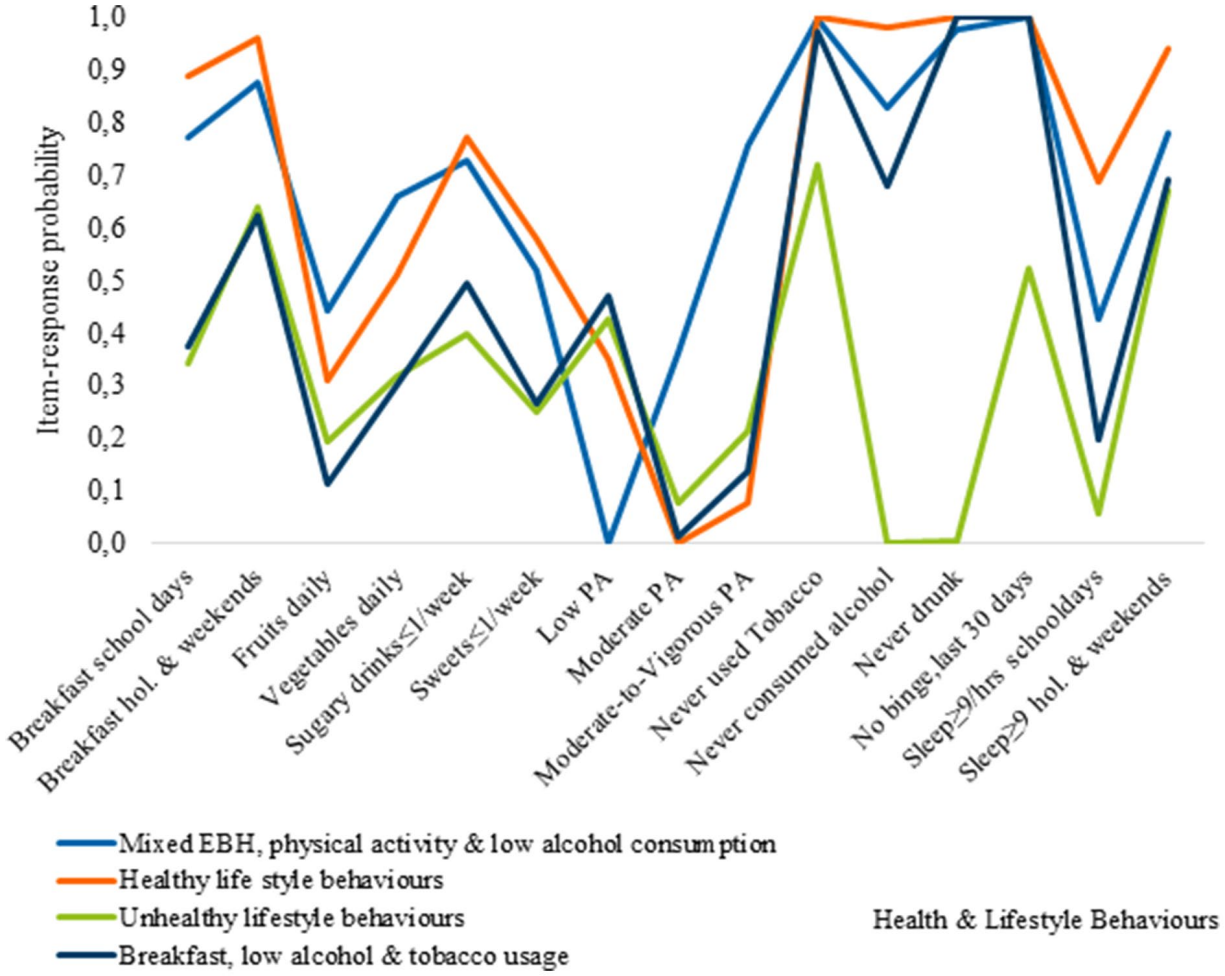
Item-response probabilities across each of the clusters for girls.

Cluster 1, represents the Mixed EBH, PA, and low AC cluster, and was characterized by adolescents with relatively healthy behaviors. They have a high probability of eating breakfast, consuming moderate amounts of vegetables, and meeting sleep recommendations on non-school days. In contrast, members of this cluster are less likely to consume sugary soft drinks, sweets, alcohol, or use tobacco. However, they engage less in PA at school but participate more in vigorous PA during their leisure time. Additionally, cluster members have a low probability of consuming fruits on a daily basis and meeting sleep recommendations on school days. Regarding breakfast consumption, boys and girls in the cluster exhibit similar healthy behaviors, although there are some sex-based differences. Girls have a higher probability than boys of consuming fruits and vegetables daily and a lower probability of consuming sugary soft drinks and sweets. Cluster 2, represents the healthy lifestyle behaviors cluster. Adolescents in this cluster had a higher likelihood of engaging in positive behaviors, such as consistent breakfast consumption, moderate levels of PA, and low rates of tobacco and alcohol use. Most of them also met sleep recommendations. There were sex-related differences in their behaviors. Boys had higher daily consumption of fruits and vegetables, as well as higher engagement in vigorous PA. In contrast, girls exhibited lower participation in vigorous physical activity (VPA) and a significant portion, approximately 35%, reported engagement in low levels of physical activity. Cluster 3, represents the unhealthy lifestyle behaviors cluster, where the majority of adolescents were more likely to engage in negative HLBs. There were some exceptions, such as a relatively high probability of eating breakfast on school days and weekends, meeting sleep recommendations on non-school days as well as, lower rates of binge drinking among boys, and tobacco use among girls.

Cluster 4, the breakfast, low AC, and TU cluster, was characterized by unhealthy eating behaviors and low PA levels, with notable sex differences in breakfast consumption. Boys had higher rates of breakfast consumption compared to girls on both school days and non-school days, although the difference was reduced on non-school days.

### Comparison of the clusters by age

The LCA age-stratified models revealed three clusters based on prominent item-response probabilities: Cluster 1 - “Unhealthy lifestyle behaviors,” Cluster 2 - “Moderately healthy lifestyle behaviors,” and Cluster 3 - “Healthy lifestyle behaviors.” Among 11-year-olds, the proportions in each cluster showed a gradient: 21% in the unhealthy cluster, 35.2% in the moderately healthy cluster, and 43.8% in the healthy cluster. For 13-year-olds, the distribution was 29.4% unhealthy, 16.2% moderately healthy, and 54.4% healthy. Among 15-year-olds, 22% were in the unhealthy cluster, while the majority (43%) were moderately healthy, and approximately 35% were healthy.

**Figure 3:**
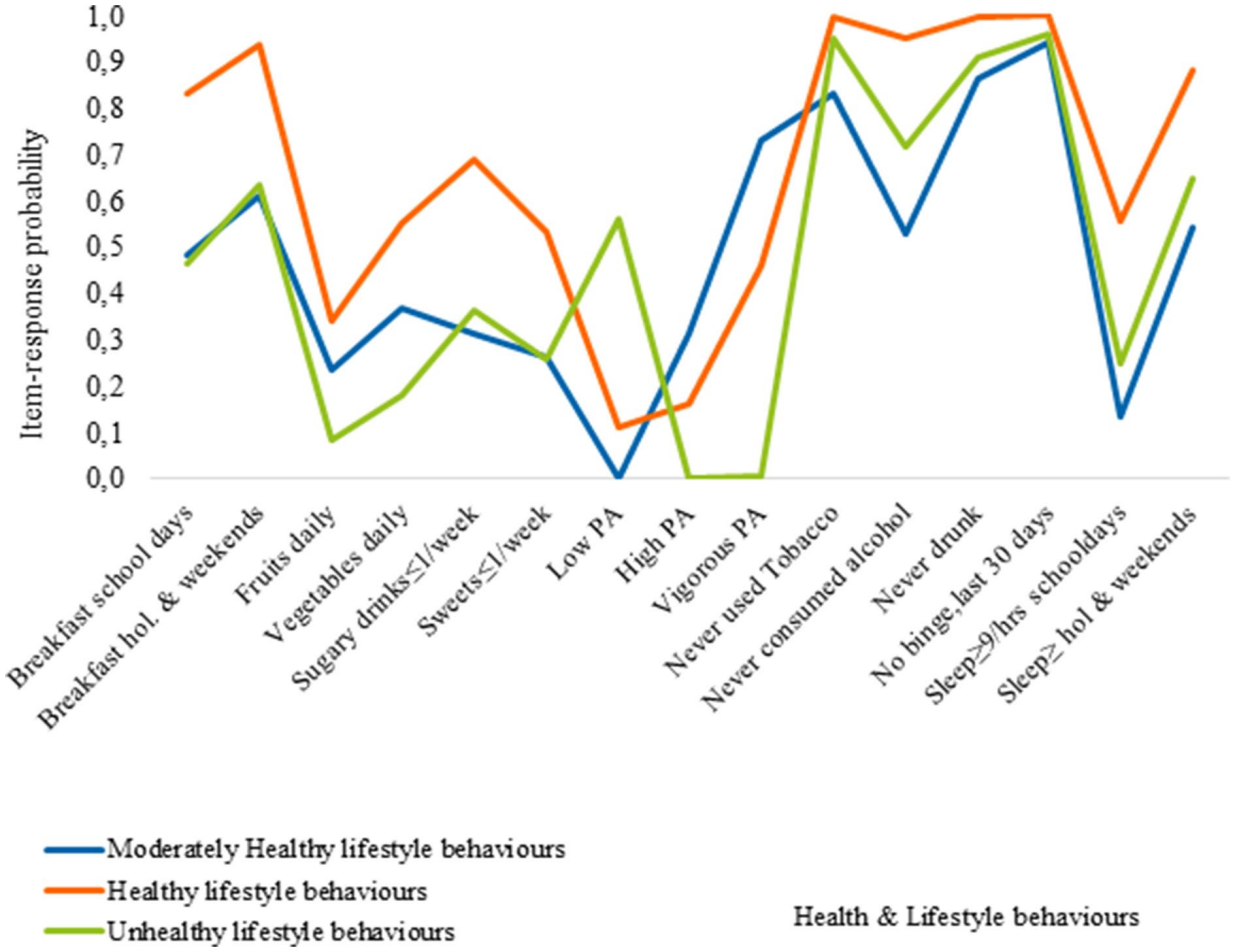
Item-response probabilities across each of the clusters for 11 year olds.

Figures 3^5^ show the item-response probabilities categorized by age. Cluster 1, representing the healthy lifestyle behaviors cluster, showed a high level of engagement in positive behaviors, such as consuming fruits and vegetables daily, and eating breakfast. The consumption of sugary drinks remained consistent across age groups, while the likelihood of eating sweets once a week or less decreased with age. Notable differences were observed between 11-year-olds and older age groups in terms of meeting sleep recommendations on school days and alcohol consumption. Older adolescents had a lower likelihood of meeting sleep recommendations, and 15-year-olds were most likely to have consumed alcohol compared to younger age groups.

**Figure 4:**
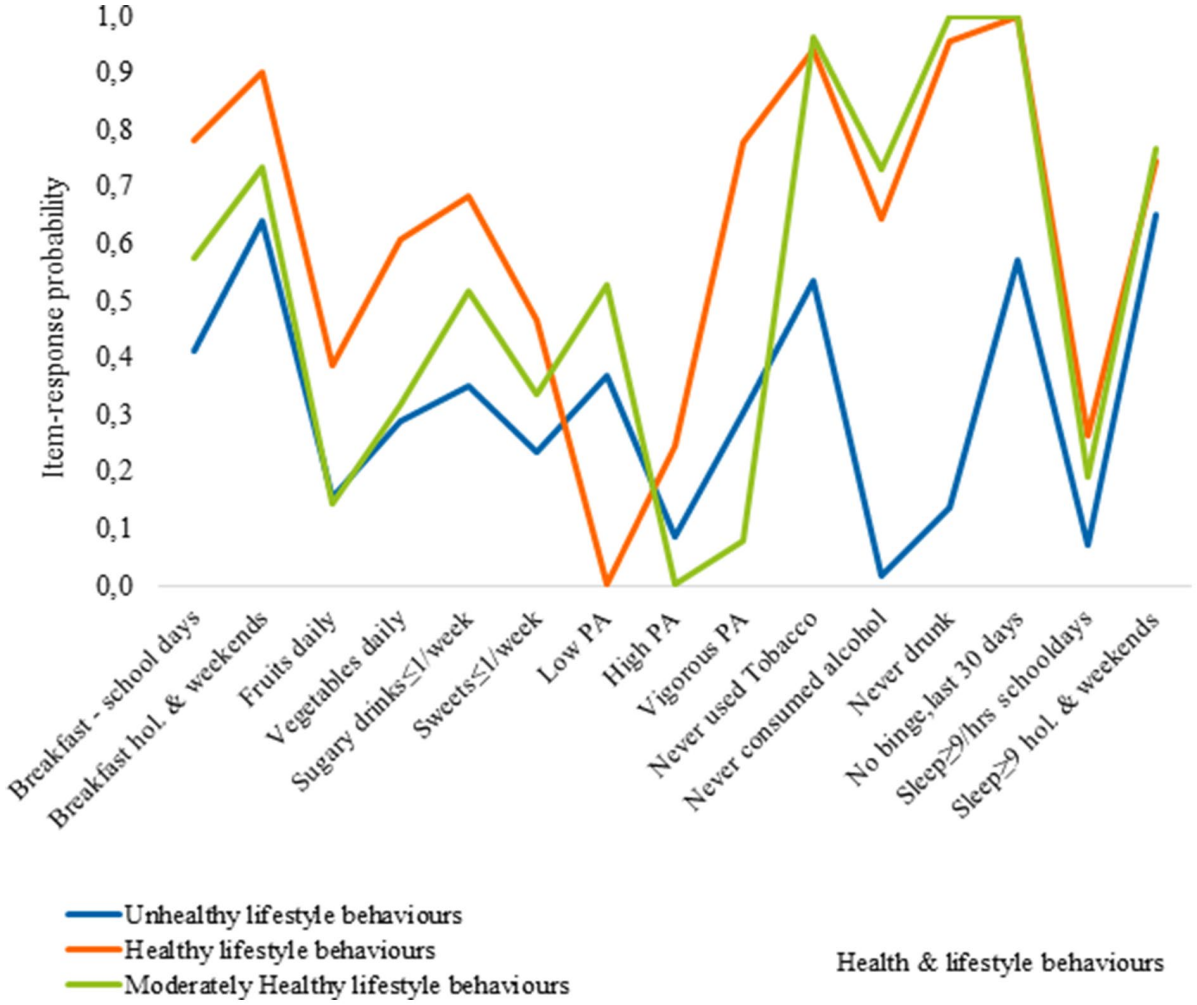
Item-response probabilities across each of the clusters for 13 year olds.

**Figure 5:**
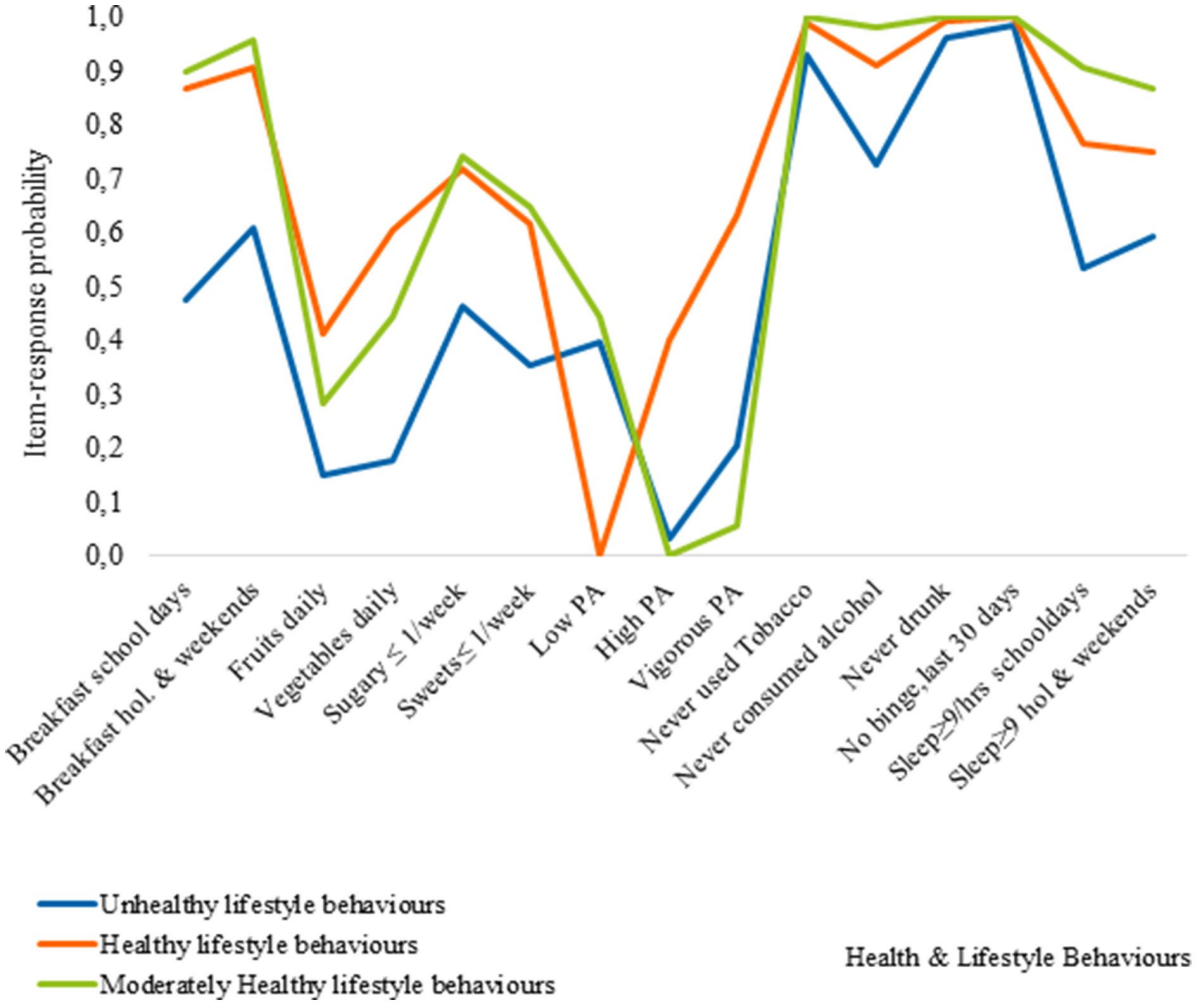
Item-response probabilities across each of the clusters for 15 year olds.

Cluster 2, represents the moderately healthy lifestyle behaviors cluster. Adolescents in this cluster had a high likelihood of consuming breakfast, with age differences observed in breakfast consumption patterns. They had lower rates of fruit and vegetable consumption and engagement in physical activity compared to the healthy lifestyle behaviors cluster. Additionally, the likelihood of meeting sleep recommendations on school days decreased with age, with 11-year-olds showing the highest rates compared to 13 and 15-year-olds.

Cluster 3, known as the unhealthy behaviors cluster, is characterized by poor EBH and low levels of PA among adolescents of all ages. However, 15-year-olds within this cluster exhibit the highest likelihood of engaging in multiple negative HLBs. Compared to other clusters, adolescents classified Cluster 3 has lower probabilities for healthy engagement across the five HLBs examined. For example, 11-year-olds in this cluster are less likely to eat breakfast on weekends compared to those in the healthy lifestyles cluster. Similar patterns are observed for other HLBs, with 13-year-olds more likely to engage in healthy behaviors, including breakfast consumption on weekends and meeting sleep recommendations on non-school days. Among 15-year-olds, a higher probability of engaging in positive HLBs is observed, such as breakfast consumption on weekends, low tobacco use, and meeting sleep recommendations on non-school days, along with a higher engagement in physical activity.

### Socioeconomic and demographic correlates of cluster membership

**Figure 6:**
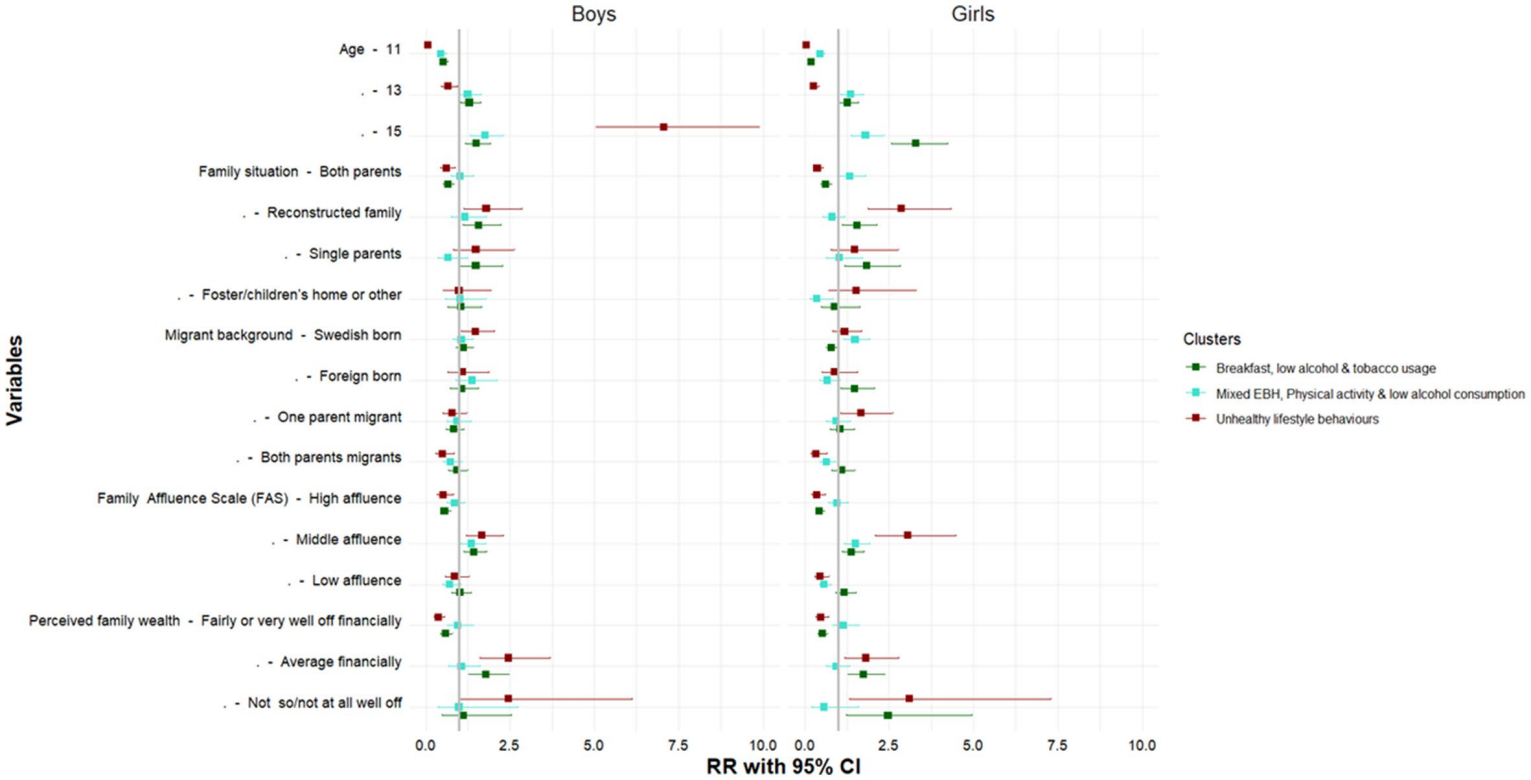
Socioeconomic and demographic correlates of each cluster by sex. Authors analysis, 2017/18 Swedish HBSC study; Exponentiated coefficients, significance *p < 0.05, **p < 0.01, ***p < 0.001; The reference cluster in the multinomial logistic regression model is the healthy behaviors clusters and the first category of each indicator.

Figures 6 7 present the results of the sex and age-stratified multinomial regression models (full results shown in Supplementary Table S1, S3), these assessed the socioeconomic and demographic correlates associated with cluster membership. Compared to the healthy lifestyle behaviors cluster, common risk factors across the other three sex-specific health clusters included age, being Swedish born, family situation, medium family affluence and low perceived family wealth. These associations varied by sex and across the HLB clusters.

For boys, only age and having medium family affluence was associated with classification in the Mixed EBH, physical activity and low alcohol consumption cluster. On the other hand, 15-year-old girls had a three times greater risk of being classified in the unhealthy behaviors cluster compared to boys of the same age. Furthermore, associations with socioeconomic circumstances were stronger for girls than boys. In particular, girls with medium family affluence and low perceived family wealth were most likely to be classified in the unhealthy lifestyle behaviors cluster. Similar risk factors were associated with classification in the Breakfast, low alcohol and tobacco usage cluster, but these effects were less pronounced. However, it is worth noting that girls in this cluster were more likely to come from single-parent households and to be foreign-born (Figure 6).

**Figure 7:**
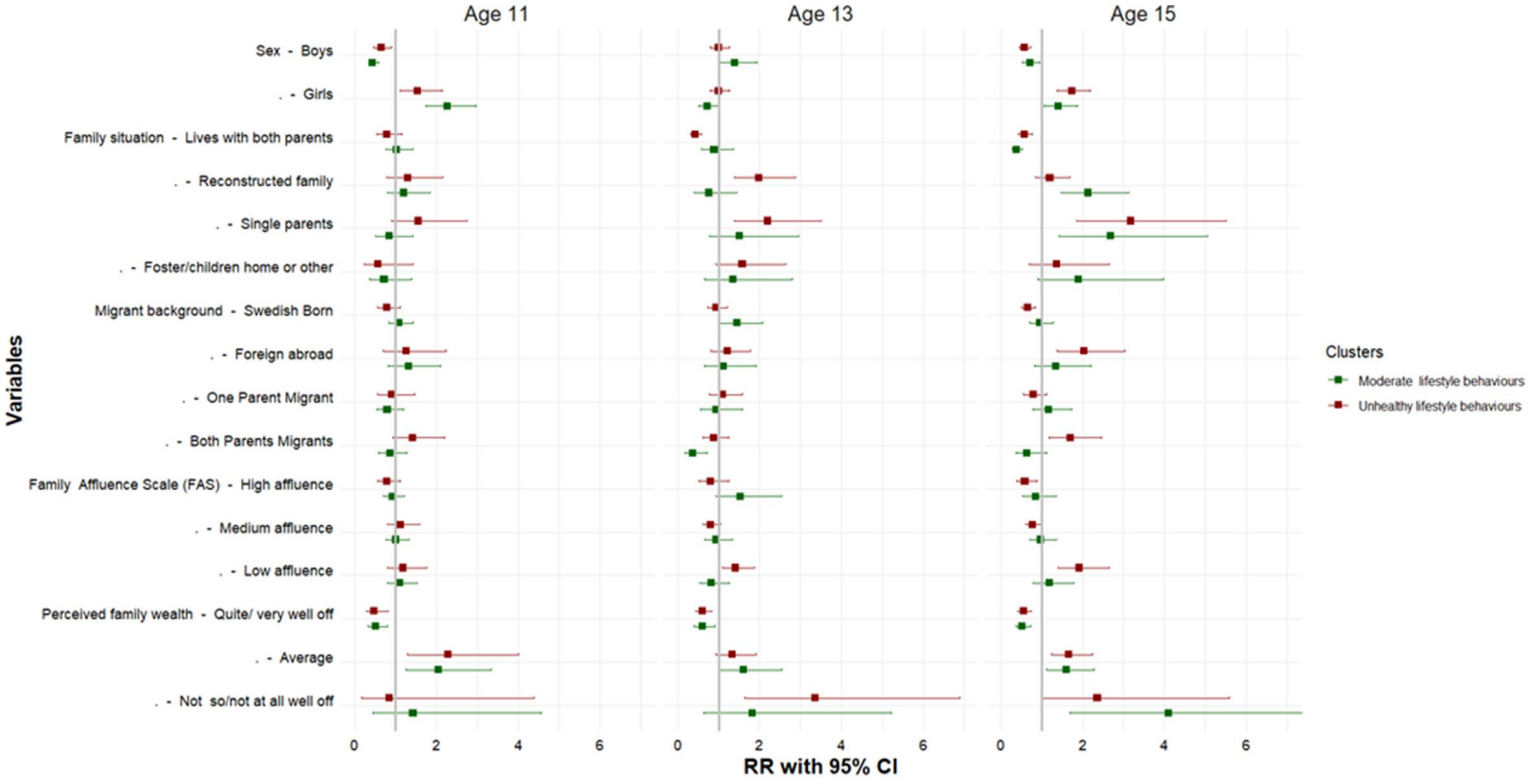
Socioeconomic and demographic correlates of each LCA by age. Authors analysis, 2017/18 Swedish HBSC study; Exponentiated coefficients, significance * *p* < 0.05, **p < 0.01, ***p < 0.001; The reference cluster in the multinomial logistic regression model is the healthy behaviors clusters and the first category of each indicator.

In the assessment of the association between cluster membership and the socioeconomic factors which may explain this relationship by age, 11-year-olds and membership in the healthy behaviors cluster were the reference categories. As such, we find that among 13-year-olds and 15-year-olds, girls and adolescents from households with average perceived family wealth had a higher risk of being classified in the moderate and unhealthy behavior clusters. While the factors related to moderate behaviors were less clear. The key socioeconomic and demographic indicators that were associated with classification in the unhealthy behavior clusters was related to migrant status and family structure. Among 13-year-olds, being Swedish born, residing in reconstructed families and single-parent household was associated with a higher risk of being classified in the unhealthy cluster. There was a similarly high risk of classification in unhealthy behavior cluster among 15-year-olds, who were foreign-born or who has two migrant parents, among those residing in reconstructed families and single-parent households 15-year-olds from had a higher risk of being classified in the unhealthy behaviors cluster. However, unlike the other clusters and age groups, 15-year-olds who were foreign-born, had low family affluence, or had two migrant parents were also more likely to be classified in the unhealthy behavior cluster (Figure 7).

## Discussion

To our knowledge, this is the first study to utilize a large nationally representative sample of adolescents, to examine multiple HLBs in the Swedish context. We showed that HLBs clustered by sex and age. Four distinct behavioral patterns were identified in the sex-stratified LCA models, while the age-stratified models pointed to three distinct clusters. Our examination of measurement invariance - aimed at assessing whether the measures of health behaviors were consistently interpreted across various groups- indicated that despite differences by age and sex, the adolescents interpreted the questions in a similar way. This finding ensures the validity and reliability of the assessment. Our study results echo the findings from earlier studies (20, 22, 23, 25, 43–45), which suggests that the frequency, intensity and overall HLB patterns are not the same for all groups (Figure 7).

Our analysis suggests that significant differences exist in health behaviors, given that boys were overrepresented in every HLB cluster except the healthy behaviors cluster. This finding is consistent with that of earlier studies (22, 43, 44). In particular, we found that, compared to girls, boys were more likely to meet breakfast recommendations but were less likely to meet other dietary requirements such as the intake of fruits, vegetables and the consumption of sugar sweetened beverages and sweets. However, our results differed from at least one study, which found that it was more common for girls to be classified in clusters engaging in poor dietary behaviours compared to boys (23). On the other hand, consistent with prior studies (23, 25, 43), our results showed that boys consistently reported greater engagement in PA, and that it was more common for them to use tobacco and drink alcohol. These results are consistent with other studies. For instance, a recent study by Miranda et al. (46) found that adolescent girls were most likely to be classified in HLB clusters characterized by a high degree of physical inactivity and sedentary behaviors. SHP also varied by sex and across all the clusters.

Furthermore, we discovered that the patterning of HLBs could be attributed to age, revealing a distinct gradient. Specifically, when comparing 11-year-olds and 15-year-olds, a clear distinction in health behaviors emerged. It was observed that the majority of 11-year-olds were classified within the healthy behaviors cluster. However, a higher percentage of 13-year-olds exhibited a slightly higher risk of being classified in the unhealthy behaviors cluster,in contrast to 15-year-olds. Such a finding would be more in line with the existing literature. For instance, a systematic review conducted by Leech et al. (43), which included 18 individual studies investigating the clustering of multiple health behaviors, reported significant age gradients in health behaviors, which suggested that older children/adolescents tend to exhibit clusters with less healthy behaviors. Despite, the slight deviation, our results are in line with increasing evidence which suggests that health behaviors formed in childhood and adolescence tracks into later life (8–12, 46).

With regard to the second objective of this study, which aimed to examine how the identified clusters relate to the socioeconomic and demographic characteristics of adolescents and their parents, we found that age, sex, family situation, migrant status, and the socioeconomic status of families were consistent predictors of cluster membership. We further observed a negative association between socioeconomic circumstances and cluster membership classification, with adolescents from more disadvantaged backgrounds being more likely to be classified in the unhealthy behaviors cluster (45, 46). However, the strength of the association found between socioeconomic status and cluster membership, varied with the measure of SES used. For example, FAS which is an absolute measure of SES was more weakly associated with cluster membership when compared to perceived family wealth, which is a relative measure of wealth.

Our study results also indicated some similarities in the factors associated with cluster membership for boys and girls. We however found stronger associations between the socioeconomic and demographic indicators and unhealthy behaviors for boys. These findings align with at least one previous study (47). The significance of these findings is underscored by previous research demonstrating the influence of socioeconomic and demographic factors on HLBs. In particular, prior studies have shown for example, that unhealthy behaviors coupled with poor socioeconomic circumstances increases the risk of morbidity and mortality (3, 16, 22, 24, 43).

### Strengths and limitations

This study has some limitations. First, the key measures used in the analyzes were based on self-reported measures with different reference periods, and they may therefore be subject to recall bias. Secondly, there are several limitations associated with the use of LCA models. LCA models employ multiple fit indices that depend on specific assumptions about the underlying statistical model. However, there is a lack of universally agreed-upon standards for their interpretation and application. Although the use of multiple indices enables a more comprehensive evaluation of the models, it is important to consider their limitations.

Furthermore, comparing the results of LCA models across studies can be challenging. This is due to variations in the identified clusters, which are determined by specific sets of items and the operationalization of those items. The number of items used in the models also varies across studies, further complicating the comparison of results (22, 24, 25, 43, 44).

Another limitation is the fact that the HBSC study aims to facilitate research on the overall health and well-being of adolescents (32). Consequently, the survey does not provide an in-depth analysis of specific nutritional intake. The questionnaire collected limited information on the frequency of intake, specifically regarding fruits, vegetables, sugar-sweetened beverages, and sweets. However, it did not capture data on the amount or portion sizes of these consumed items. Despite this limitation, the instrument is widely used and considered a valid measure of the dietary patterns of adolescents (31, 32, 48–50).

Notwithstanding these limitations, a strength of the current study was the inclusion of self-reported data obtained from children and adolescents participating in the HBSC study. This enabled the study to gather their unique perspectives and experiences regarding living conditions and health behaviors. Notwithstanding these limitations, a strength of the current study was the inclusion of self-reported data obtained from children and adolescents participating in the HBSC study. This enabled the study to gather their unique perspectives and experiences regarding living conditions and health behaviors.

An advantage of using LCA is the possibility to examine the inter/intra-item relationships between multiple health behaviors simultaneously, and to assess numerous combinations of unobserved behavior patterns before reducing the data into smaller clusters (51). This current analyzes therefore contributes to our understanding of the interlinkage between various health behaviors while allowing for assessments of the differences in health behaviors by factors such as age and sex. Moreover, the use of LCA contributes to the growing scientific literature suggesting that health behaviors are not randomly distributed, but rather cluster within individuals and across similar socioeconomic and demographic characteristics (19, 24, 25, 28).

## Conclusion

This study identifies distinct clusters based on sex and age and demonstrates that these clusters correlate with the socioeconomic and demographic characteristics of both adolescents and their parents. Many health-focused policy initiatives and programs tend to narrowly target specific lifestyle behaviors like physical activity or eating habits. However, our findings emphasize the importance of implementing more effective and targeted interventions that prioritize overall behavioral change among vulnerable groups and at-risk adolescents. Future research should investigate the effectiveness of employing a multi-behavioral change approach in prevention strategies.

## Data availability statement

The data analyzed in this study is subject to the following licenses/restrictions: Requests to access these datasets should be directed to Application and enquiries for data use should be sent to skolbarns.halsovanor@folkhalsomyndigheten.se.

## Ethics statement

The studies involving human participants were reviewed and approved by The Public Health Agency of Sweden and Statistics Sweden SCB - The national statistics office of Sweden. Informed consent to participate in this study was provided by the participants’ legal guardian/next of kin.

## Author contributions

KJ: conceptualization and formal analysis. KJ and NA: methodology. KJ, PL, MC, and NA: writing–review and editing. All authors have read and agreed to the published version of the manuscript.

## Conflict of interest

The authors declare that the research was conducted in the absence of any commercial or financial relationships that could be construed as a potential conflict of interest.

## Publisher’s note

All claims expressed in this article are solely those of the authors and do not necessarily represent those of their affiliated organizations, or those of the publisher, the editors and the reviewers. Any product that may be evaluated in this article, or claim that may be made by its manufacturer, is not guaranteed or endorsed by the publisher.
