# Supplementary material for: The clustering of multiple health and lifestyle behaviors among Swedish adolescents: a person-oriented analysis
Source: Front Public Health. 2023 Jul 19;11:1178353. doi: 10.3389/fpubh.2023.1178353 (PMC10394625; doi:10.3389/fpubh.2023.1178353)
Supplement: Supplementary file 1 [file Data_Sheet_1.PDF]

Supplementary Appendix: The clustering of multiple health and lifestyle behaviours among Swedish adolescents: A person-oriented analysis

Kenisha Russell Jonsson[1], Maria Corell[1], Petra Löfstedt[1], Nicholas Kofi Adjei[234]

[1] School of Public Health and Community Medicine, Institute of Medicine, Gothenburg University, Box 463, 405 30, Göteborg, Sweden

[2] University of Liverpool, Department of Public Health and Policy, Waterhouse Building 2nd Floor , Block F, Liverpool , L69 3GL, UK

[3] Leibniz Institute for Prevention Research and Epidemiology - BIPS, Bremen, Germany

[4] Health Sciences Bremen, University of Bremen, Bremen, Germany

Supplementary Table 1. Goodness of fit statistics for LCA models by sex and age (Unconstrained)

SEX					
**Number of clusters**	**DF**	**G2**	**AIC**	**BIC**	**ENTROPY**
**2**	65473	8734	8858	9247	0.86
**3**	65441	7391	7579	8169	0.71
**4**	**65409**	**6757**	**7009**	**7800**	**0.70**
**5**	65377	6502	6818	7810	0.69
**6**	65345	6178	6558	7751	0.68
**AGE**					
**Number of clusters**	**DF**	**G2**	**AIC**	**BIC**	**ENTROPY**
**2**	98210	9103	9289	9873	0.69
**3**	**98162**	**8015**	**8297**	**9182**	**0.68**
**4**	98114	7490	7868	9054	0.70
**5**	98066	7174	7648	9136	0.69
**6**	98018	6846	7416	9206	0.69

Notes. *DF= degrees of freedom, G2= likelihood ratio chi-square. Items in **bold** represented chosen model,All Parameters Estimated Freely/Unconstrained.

Supplementary Table 1. shows the degrees of freedom , G2 test statistic and information criteria/fit indices used to assess the adequacy of the final model. Examination of the model solutions indicated a model with four and three clusters, respectively had the best realative fit based on the AIC, BIC and entropy. In addition, the selected models were (1) identified (2) showed parsimony; (3) had a sufficient sample sizes in each identified cluster; and (4) the parameter estimates presented a solution that could be both logically and substantively interpreted.

Measurement invariance

Measurement invariance (MI), which is a method of evaluating how well a specified model fits the observed data, was used to assess model constraint. For the Sex-stratified models, one model in which all parameters were constrained to be equal for boys and girls. This was followed by a model with all parameters freely estimated (that is, an unconstrained model). The G^2 statistic for the unconstrained model was 6757 with 65

409 degrees of freedom *(df; AIC* =7009) and 6950 with 65 452 degrees of freedom (*df; 7116.38*) for the constrained model. This resulted in a likelihood-ratio statistic G^2 difference of 445 (*df* =60). This difference is non-significant, demonstrating MI by sex which suggests that the clusters have a similar meaning for boys and girls.

An assessment of MI for models fitted by age resulted in a non-significant likelihood-ratio of 1115 (df = 90). This was based on the difference between the G2 statistic for the unconstrained model [5572 (df = 98162)] and the constrained model [6687 (df = 98252)]. The results indicated that MI holds; therefore the clusters were comparable across the three age groups.

The results from the unconstrained models were reported because the AIC and BIC suggested that the unconstrained models were preferable. Moreover, the results from unconstrained models provides differences in the item-response probabilities and this may therefore shed light on the nature and extent of the probable underlying sex and age differences.

Supplementary figure 1a & Figure 1b. Cluster membership probabilities by sex and age

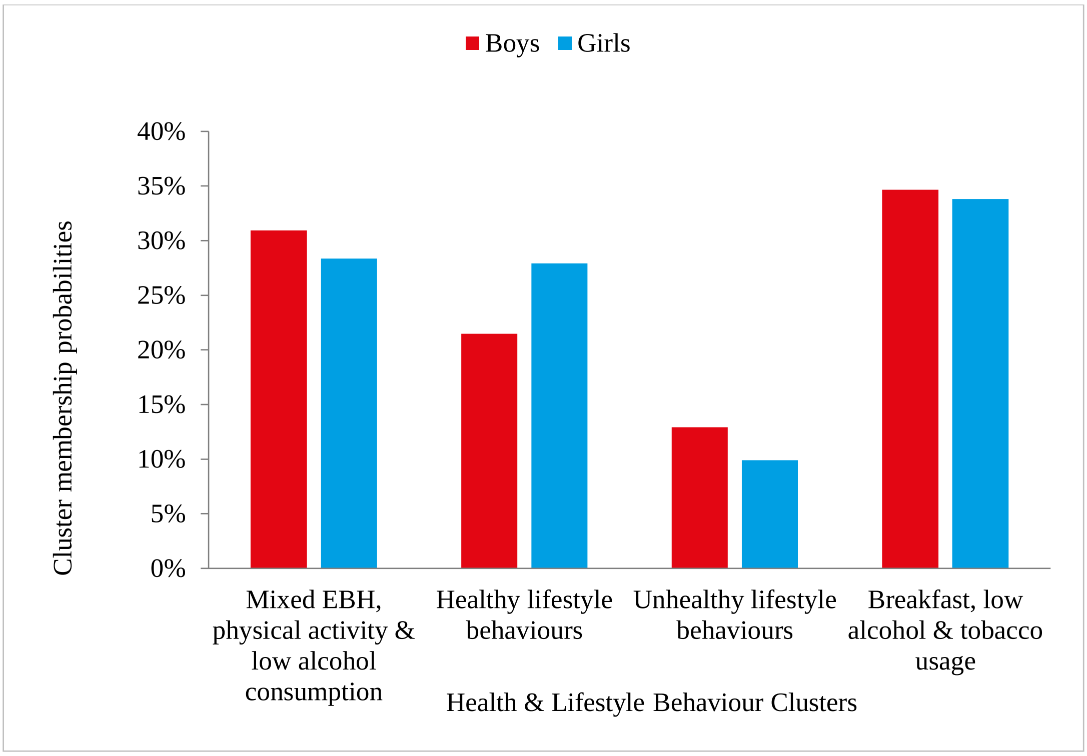

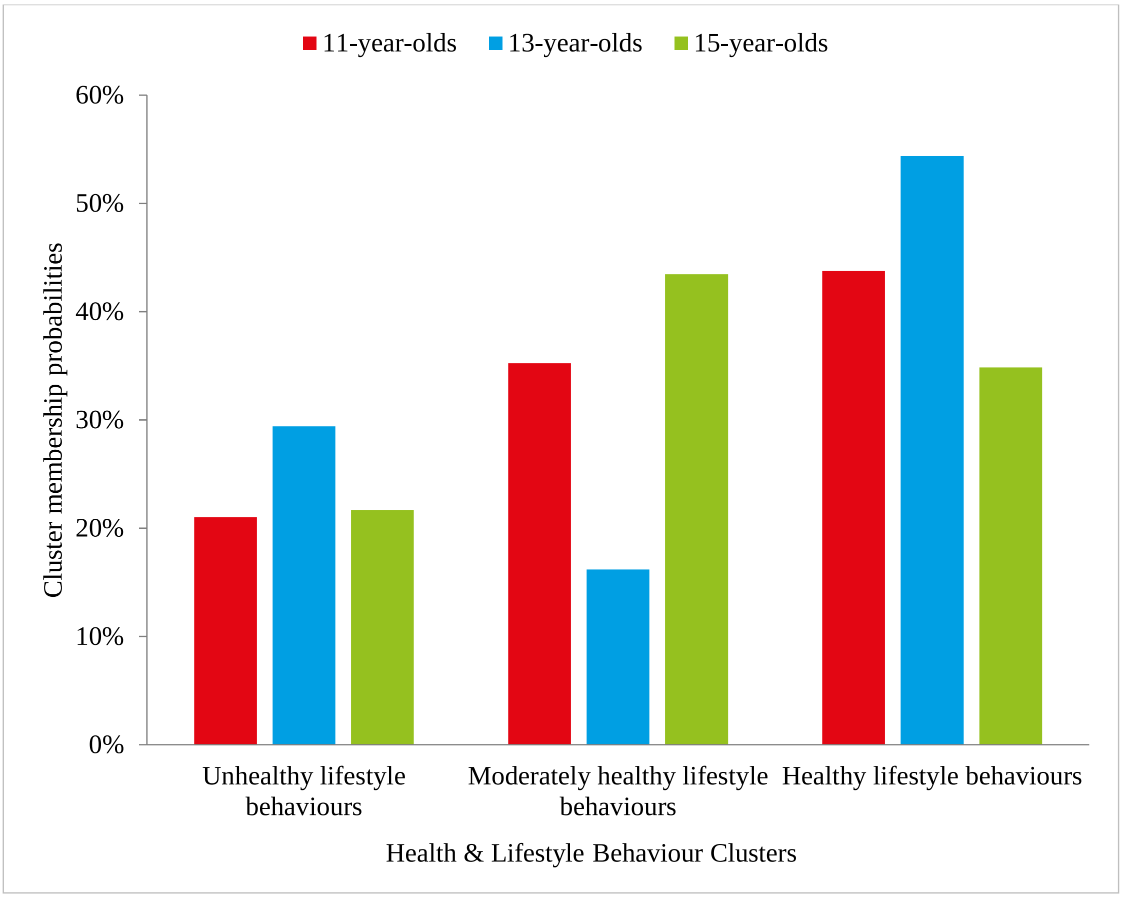

Class membership is the estimated class, in which each adolescent person belongs to (i.e., what type of health and lifestyle behaviour each person has). For a given adolescent, PROC LCA estimates the probability that they belong to each given class (the first, second, third and/or the fourth class.

Supplementary Table 2. Socioeconomic and demographic correlates of each LCA by sex

	**Boys**									**Girls**								
	**Healthy lifestyle behaviours**		**Mixed EBH, Physical activity & low alcohol consumption**		**Unhealthy lifestyle behaviours**		**Breakfast, low alcohol & tobacco usage**			**Healthy lifestyle behaviours**		**Mixed EBH, Physical activity & low alcohol consumption**		**Unhealthy lifestyle behaviours**		**Breakfast, low alcohol & tobacco usage**		
	**Odds Ratio**	**CI**	**Odds Ratio**	**CI**	**Odds Ratio**	**CI**	**Odds Ratio**	**CI**	**No. of obs in model**	**Odds Ratio**	**CI**	**Odds Ratio**	**CI**	**Odds Ratio**	**CI**	**Odds Ratio**	**CI**	**No. of obs in model**
**Age**																		
11	Ref.	Ref.	0.46***	[0.34,0.61]	0.07***	[0.04,0.13]	0.53***	[0.42,0.66]	1939	Ref.	Ref.	0.45***	[0.35,0.58]	0.04***	[0.02,0.08]	0.21***	[0.17,0.28]	1998
13	Ref.	Ref.	1.25	[0.96,1.64]	0.67*	[0.47,0.94]	1.30*	[1.03,1.63]		Ref.	Ref.	1.36*	[1.06,1.74]	0.26***	[0.16,0.42]	1.26	[1.00,1.59]	
15	Ref.	Ref.	1.76***	[1.33,2.31]	7.07***	[5.05,9.90]	1.51***	[1.19,1.91]		Ref.	Ref.	1.80***	[1.38,2.36]	23.00***	[14.63,36.18]	3.30***	[2.57,4.23]	
**Family situation**																		
Both parents	Ref.	Ref.	1.03	[0.74,1.42]	0.62**	[0.43,0.88]	0.66**	[0.51,0.85]	1716	Ref.	Ref.	1.33	[0.98,1.80]	0.37***	[0.26,0.54]	0.61***	[0.47,0.80]	1791
Reconstructed family	Ref.	Ref.	1.17	[0.75,1.80]	1.80*	[1.13,2.86]	1.58*	[1.11,2.24]		Ref.	Ref.	0.80	[0.54,1.18]	2.86***	[1.88,4.34]	1.54**	[1.11,2.13]	
Single parents	Ref.	Ref.	0.66	[0.36,1.23]	1.49	[0.83,2.64]	1.49	[0.98,2.27]		Ref.	Ref.	1.03	[0.62,1.71]	1.47	[0.78,2.77]	1.83**	[1.19,2.83]	
Foster/children’s home or other	Ref.	Ref.	1.03	[0.59,1.81]	0.99	[0.50,1.94]	1.04	[0.65,1.66]		Ref.	Ref.	0.36*	[0.15,0.84]	1.53	[0.70,3.31]	0.89	[0.49,1.64]	
**Migrant background**																		
Swedish born	Ref.	Ref.	1.06	[0.81,1.39]	1.48*	[1.07,2.05]	1.12	[0.90,1.41]	1939	Ref.	Ref.	1.49**	[1.16,1.92]	1.18	[0.83,1.67]	0.78*	[0.62,0.98]	1998
Foreign born	Ref.	Ref.	1.38	[0.90,2.11]	1.10	[0.65,1.86]	1.07	[0.73,1.56]		Ref.	Ref.	0.67	[0.44,1.03]	0.89	[0.51,1.55]	1.48*	[1.05,2.07]	
One parent migrant	Ref.	Ref.	0.93	[0.64,1.34]	0.78	[0.50,1.23]	0.83	[0.61,1.13]		Ref.	Ref.	0.92	[0.63,1.34]	1.67*	[1.06,2.61]	1.04	[0.74,1.47]	
Both parents migrants			0.74	[0.50,1.09]	0.50*	[0.29,0.85]	0.92	[0.67,1.25]				0.64*	[0.44,0.92]	0.33**	[0.17,0.66]	1.10	[0.81,1.50]	
**Family Affluence Scale (FAS)**																		
High affluence	Ref.	Ref.	0.85	[0.63,1.16]	0.53**	[0.35,0.81]	0.56***	[0.43,0.75]	1876	Ref.	Ref.	0.95	[0.71,1.27]	0.36***	[0.21,0.61]	0.43***	[0.31,0.59]	1940
Medium affluence	Ref.	Ref.	1.36*	[1.04,1.78]	1.66**	[1.20,2.29]	1.43**	[1.14,1.79]		Ref.	Ref.	1.50**	[1.17,1.91]	3.05***	[2.09,4.47]	1.39**	[1.11,1.75]	
Low affluence	Ref.	Ref.	0.72	[0.51,1.03]	0.87	[0.58,1.30]	1.03	[0.78,1.35]		Ref.	Ref.	0.58***	[0.43,0.78]	0.46***	[0.29,0.73]	1.17	[0.91,1.51]	
**Perceived family wealth**																		
Quite/very well off	Ref.	Ref.	0.95	[0.64,1.42]	0.39***	[0.26,0.57]	0.59***	[0.43,0.80]	1903	Ref.	Ref.	1.15	[0.82,1.62]	0.47***	[0.32,0.70]	0.52***	[0.39,0.69]	1950
Average	Ref.	Ref.	1.06	[0.69,1.62]	2.45***	[1.62,3.69]	1.78***	[1.28,2.48]		Ref.	Ref.	0.92	[0.64,1.32]	1.81**	[1.18,2.77]	1.74***	[1.28,2.37]	
Not so/not at all well off	Ref.	Ref.	0.99	[0.36,2.74]	2.45	[0.98,6.11]	1.11	[0.48,2.55]		Ref.	Ref.	0.58	[0.21,1.59]	3.11**	[1.33,7.30]	2.47*	[1.23,4.96]	

Source: 2017/18 Swedish HBSC study;

Notes: Exponentiated coefficients; 95 % confidence intervals in brackets * p<0.05, ** p<0.01, *** p<0.001. For each row , Ref. indicates the reference cluster in the multinomial logistic regression model

Supplementary Table 3. Socioeconomic and demographic correlates of each LCA by age

11-year-olds / 13-year-olds

	11-year-olds: Healthy lifestyle behaviours		11-year-olds: Moderate lifestyle behaviours		11-year-olds: Unhealthy lifestyle behaviours			13-year-olds: Healthy lifestyle behaviours		13-year-olds: Moderate lifestyle behaviours		13-year-olds: Unhealthy lifestyle behaviours		
	Odds Ratio	CI	Odds Ratio	CI	Odds Ratio	CI	No. Of obs in model	Odds Ratio	CI	Odds Ratio	CI	Odds Ratio	CI	No. Of obs in model
**Sex**														
Boys	Ref.	Ref.	0.44***	[0.34,0.58]	0.65*	[0.47,0.90]	1110	Ref.	Ref.	1.39	[0.99,1.93]	1.00	[0.79,1.27]	1330
Girls	Ref.	Ref.	2.27***	[1.74,2.97]	1.53*	[1.11,2.13]		Ref.	Ref.	0.72	[0.52,1.01]	1.00	[0.79,1.27]	
**Family situation**														
Lives with both parents	Ref.	Ref.	1.03	[0.75,1.42]	0.79	[0.54,1.16]	995	Ref.	Ref.	0.89	[0.58,1.36]	0.43***	[0.33,0.58]	1168
Reconstructed family	Ref.	Ref.	1.21	[0.79,1.84]	1.30	[0.78,2.16]		Ref.	Ref.	0.77	[0.40,1.45]	1.99***	[1.38,2.87]	
Single parents	Ref.	Ref.	0.84	[0.50,1.42]	1.56	[0.89,2.74]		Ref.	Ref.	1.51	[0.77,2.96]	2.20***	[1.38,3.50]	
Foster/children home or other	Ref.	Ref.	0.72	[0.37,1.39]	0.57	[0.23,1.43]		Ref.	Ref.	1.35	[0.65,2.80]	1.58	[0.94,2.65]	
**Migrant background**														
Swedish born	Ref.	Ref.	1.09	[0.83,1.43]	0.79	[0.57,1.11]		Ref.	Ref.	1.45*	[1.01,2.08]	0.94	[0.74,1.21]	1330
Foreign born	Ref.	Ref.	1.32	[0.82,2.10]	1.26	[0.71,2.24]	1110	Ref.	Ref.	1.12	[0.66,1.91]	1.21	[0.82,1.78]	
One parent migrant	Ref.	Ref.	0.80	[0.54,1.19]	0.91	[0.56,1.46]		Ref.	Ref.	0.94	[0.56,1.58]	1.10	[0.77,1.58]	
Both parents migrants	Ref.	Ref.	0.87	[0.59,1.28]	1.42	[0.93,2.19]		Ref.	Ref.	0.37**	[0.19,0.72]	0.88	[0.61,1.25]	
**Family Affluence Scale (FAS)**														
High affluence	Ref.	Ref.	0.93	[0.71,1.22]	0.79	[0.56,1.11]	1059	Ref.	Ref.	1.53	[0.93,2.54]	0.80	[0.51,1.25]	1289
Medium affluence	Ref.	Ref.	1.01	[0.76,1.33]	1.13	[0.80,1.58]		Ref.	Ref.	0.94	[0.66,1.35]	0.80	[0.62,1.04]	
Low affluence	Ref.	Ref.	1.10	[0.79,1.53]	1.18	[0.79,1.76]		Ref.	Ref.	0.83	[0.54,1.26]	1.42*	[1.08,1.88]	
**Perceived family wealth**														
Quite/very well off	Ref.	Ref.	0.51**	[0.32,0.80]	0.48**	[0.28,0.82]	1085	Ref.	Ref.	0.60*	[0.39,0.92]	0.60**	[0.43,0.83]	1297
Average	Ref.	Ref.	2.05**	[1.26,3.33]	2.28**	[1.30,4.00]		Ref.	Ref.	1.61*	[1.01,2.54]	1.34	[0.93,1.92]	
Not so/not at all well off	Ref.	Ref.	1.43	[0.45,4.55]	0.84	[0.16,4.39]		Ref.	Ref.	1.82	[0.63,5.22]	3.36***	[1.63,6.89]	

Source: Authors analysis, 2017/18 Swedish HBSC study;

Notes: Exponentiated coefficients; 95 % confidence intervals in brackets * p<0.05, ** p<0.01, *** p<0.001. For each row, **Ref.** indicates the reference cluster in the multinomial logistic regression model

15-year-olds

	Healthy lifestyle behaviours		Moderate lifestyle behaviours		Unhealthy lifestyle behaviours		
	Odds Ratio	CI	Odds Ratio	CI	Odds Ratio	CI	No. Of obs in model
**Sex**							
Boys	Ref.	Ref.	0.71*	[0.53,0.94]	0.58***	[0.46,0.73]	1497
Girls	Ref.	Ref.	1.41*	[1.07,1.87]	1.74***	[1.38,2.19]	
**Family situation**							
Lives with both parents	Ref.	Ref.	0.38***	[0.28,0.53]	0.58***	[0.43,0.76]	1344
Reconstructed family	Ref.	Ref.	2.14***	[1.47,3.12]	1.20	[0.85,1.69]	
Single parents	Ref.	Ref.	2.68**	[1.42,5.05]	3.18***	[1.84,5.51]	
Foster/children home or other	Ref.	Ref.	1.89	[0.90,3.97]	1.36	[0.69,2.65]	
**Migrant background**							
Swedish born	Ref.	Ref.	0.95	[0.70,1.28]	0.66***	[0.52,0.84]	1497
Foreign born	Ref.	Ref.	1.34	[0.82,2.21]	2.04***	[1.37,3.03]	
One Parent migrant	Ref.	Ref.	1.17	[0.79,1.73]	0.79	[0.55,1.12]	
Both Parents migrants	Ref.	Ref.	0.64	[0.37,1.12]	1.70**	[1.17,2.46]	
**Family Affluence Scale (FAS)**							
High affluence	Ref.	Ref.	0.85	[0.53,1.35]	0.59*	[0.39,0.89]	1468
Medium affluence	Ref.	Ref.	0.97	[0.70,1.35]	0.78	[0.60,1.01]	
Low affluence	Ref.	Ref.	1.19	[0.79,1.78]	1.92***	[1.40,2.65]	
**Perceived family wealth**							
Quite/very well off	Ref.	Ref.	0.52***	[0.37,0.73]	0.56***	[0.42,0.75]	1471
Average	Ref.	Ref.	1.60*	[1.12,2.28]	1.66***	[1.23,2.24]	
Not so/not at all well off	Ref.	Ref.	4.11**	[1.69,10.04]	2.36	[0.99,5.59]	

Source: Authors analysis, 2017/18 Swedish HBSC study; Notes: Exponentiated coefficients;
95 % confidence intervals in brackets * p<0.05, ** p<0.01, *** p<0.001.
For each row, Ref. indicates the reference cluster in the multinomial logistic regression model.
